# The effect of altruism on COVID-19 vaccination rates

**DOI:** 10.1186/s13561-022-00415-6

**Published:** 2023-01-03

**Authors:** Luis Á. Hierro, David Patiño, Pedro Atienza, Antonio J. Garzón, David Cantarero

**Affiliations:** 1 Department of Economics and Economic History, University of Sevilla, Avda. Ramón y Cajal, S/N, 41018 Seville, Spain; 2 Department of Economics, Universidad de Cantabria, Research Group on Health Economics and Health Services Management—Marqués de Valdecilla Research Institute (IDIVAL), Avda. de los Castros S/N, 39005 Santander, Spain

**Keywords:** Altruism, Vaccines, COVID-19; hesitancy, Externality, Herd immunity, Public health

## Abstract

**Background:**

After the emergence of the first vaccines against the COVID-19, public health authorities have promoted mass vaccination in order to achieve herd immunity and reduce the effects of the disease. Vaccination rates have differed between countries, depending on supply (availability of resources) and demand (altruism and resistance to vaccination) factors.

**Methods:**

This work considers the hypothesis that individuals’ health altruism has been an important factor to explain the different levels of vaccination between countries, using the number of transplants as a proxy for altruism. Taking European Union’s countries to remove, as far as possible, supply factors that might affect vaccination, we carry out cross-sectional regressions for the most favorable date of the vaccination process (maximum vaccination speed) and for each month during the vaccination campaign.

**Results:**

Our findings confirm that altruism has affected vaccination rates against the COVID-19. We find a direct relationship between transplants rates (proxy variable) and vaccination rates during periods in which the decision to be vaccinated depended on the individual’s choice, without supply restrictions. The results show that other demand factors have worked against vaccination: political polarization and belonging to the group of countries of the former Eastern bloc.

**Conclusions:**

Altruism is a useful tool to define future vaccination strategies, since it favors the individuals’ awareness for vaccination.

**Supplementary Information:**

The online version contains supplementary material available at 10.1186/s13561-022-00415-6.

## Background

The fight against the COVID-19 pandemic has exposed inequalities in public health. At the beginning of the pandemic, these inequalities were manifested in clinical care and, later, in vaccination against SARS-CoV-2. The determining factor in a country's vaccination capacity is income [1, 2]. High-income countries were not financially constrained and were able to access vaccines from the time they became available. In the first months of the vaccination campaign, they suffered temporary supply and logistics problems, but by the end of August 2021, vaccination had already reached rates of over 50% in most countries. However, the poorest countries—those lacking the economic capacity to acquire vaccines—experienced very slow vaccination processes with very low vaccination rates [3]. This inequality in vaccination between rich and poor countries has triggered an important ethical debate [4]. However, although rich countries have obtained vaccines—especially after the first months of vaccination—not all of them have followed the same vaccination patterns or have reached the same population vaccination rates. Previous studies show that gaps in vaccination levels are associated with disparities in income, educational level, sex and race, poverty status, etc. [5–7] and that citizens’ nonacceptance of vaccines plays an important role [8, 9].

Social controversies surrounding vaccines have been common since the first vaccines were developed [10–15]. In recent decades, antivaccine movements have grown throughout much of the world [16, 17] such that, even before vaccines against SARS-CoV-2 became available, as early as 2020, surveys and studies on citizen willingness to get vaccinated were conducted [18–22]. Many such studies on pandemics have been carried out, and there are already numerous works compiling their main results [23–26] or addressing specific groups such as health workers or students in the health field [27–30] or pregnant women [31]. These works reveal how people refuse to be vaccinated against SARS-CoV-2 for a variety of different reasons related to the following:

a) Confidence in the vaccines themselves, vaccine side effects or safety [32–38], their efficacy [32, 38–40], the need for vaccines [39], and the development process behind these medicines [41]. Factors that favour trust or receptivity have also been analysed [42].

b) The development of pandemic and antipandemic measures, the perception of the probability of contagion and severity of the disease [37, 40, 43–45], the course of the pandemic [46, 47], and the requirement of vaccination certificates for certain activities [48–51].

c) Political issues, such as lack of trust in government [19, 52], doubts about the vaccine authorization process [53, 54], conspiracy theories [33, 43, 52], ideological positioning and political polarization [15, 33, 37, 40, 43, 55–61].

The main shortcoming concerning this strand of the literature is the lack of studies that assess what impact altruism may have on the willingness to get vaccinated, since altruism is positively linked to the positive external effects of vaccination. Indeed, the main characteristics of vaccines include their positive external effects, that is, the benefits they provide for the well-being of people other than those who are vaccinated (social benefits). When an individual is vaccinated, two positive external effects occur: a direct external effect, associated with the reduced likelihood of infecting people with whom we interact, especially direct family members, and a collective external effect, which occurs as a result of our vaccination reducing the general probability of contagion. This general probability of contagion depends inversely on the percentage of people vaccinated, although in a nonlinear manner. When the percentage of those vaccinated is small, the external effects are also small. Positive external effects grow more than proportionally as the percentage of vaccinated people grows. When a certain threshold is reached in the proportion of immune individuals, the incidence of infection begins to decrease. At that moment, what is known as herd immunity is reached [62]. Acquiring herd immunity is the fundamental instrument of public health in a pandemic situation while there is no drug available to mitigate or eliminate the effects of the disease [63–66].

If we use the term *health altruism* to refer to the assessment the individual makes of the social benefits of being vaccinated, then the greater the population’s health altruism, the higher a country’s vaccination rate must be and, therefore, the greater the possibility of achieving herd immunity. This work aimed to pinpoint this effect by testing the hypothesis that social altruism favours vaccination against COVID-19 and proves decisive vis-à-vis the existence of different vaccination rates. For this purpose, the number of organ transplants was used as a proxy for health altruism, since the concept of altruism forms the emotional basis of transplant ethics widely recognized in the literature [67–72]. This means that we can consider transplants as a real manifestation of individuals’ will to contribute to the health of others and, therefore, of altruism in health matters.

The works published to date have not attempted to pinpoint the influence of health altruism on vaccination rates against COVID-19, such that there are no previous references or data from surveys. To test the hypothesis, we performed a cross-sectional analysis, taking vaccination data from EU countries. This selection was due to the fact that the EU has centrally managed the purchase of vaccines for all EU countries and has distributed them equally, substantially reducing the influence of supply factors related to the accessibility and availability of the vaccine.

Our work shows that during the first phase of vaccination when supply constraints were in effect, that is, when there were insufficient vaccines available and the shortage affected vaccination, health altruism had no impact. However, when supply restrictions disappeared and individuals’ desire to be vaccinated became a factor, health altruism did come into play as an important variable to explain countries’ different vaccination rates. Likewise, this paper shows that other demand factors negatively affect vaccination, such as being a former Eastern European Bloc country and the level of political polarization.

The structure of this work is as follows: in Methods section, we explain the methodology used and the data sources. In Results section, we show the results obtained, and in Discussion section, we comment on the results, analysing their limitations and implications. In Conclusion section, we present the conclusions.

## Methods

The vaccination process is determined not only by subjects’ willingness to be vaccinated, which manifests itself in the demand for vaccination but also by the availability of the vaccine, that is, by supply. When the government carries out vaccination, there are basically three supply factors: a) vaccines are available on the market, b) the government can pay for vaccines, and c) the government has the logistics and health system required to carry out mass vaccination. Factors b) and c) are related to the country’s economic capacity, while factor a)—the existence of available vaccines—is related to the organization of a global manufacturing system.

### Geographical scope

To minimize the impact of factors b) and c), we chose to limit this study to a restricted geographical area: the EU. The EU carried out the acquisition and distribution of vaccines to achieve equitable distribution and reduce the effects of competition between countries on the price and availability of vaccines. Furthermore, the EU also financed governments to cover the costs of the pandemic, which avoided substantial differences in distribution logistics. The result of this European intervention was that the influence of supply factors b) and c) is almost eliminated if we select this particular geographical area.

### Timeframe

For its part, factor a)—vaccine scarcity—is related to the moment. The more advanced the vaccination campaign, the smaller the effect of the vaccine shortage. Therefore, if we wait until the end of the vaccination process, we eliminate the effect of the shortage. However, this implies missing out on relevant information for public health authorities in terms of influencing the vaccination process, such that we must look for another alternative.

The problem is that choosing any other day implies a bias, which in this work we try to eliminate by using two methods: I) The first involves taking the day on which the vaccination speed is at its highest for each country; and II) the second involves making a cross-section at the end of each completed month since the start of vaccination in the EU.

For Method I), the concept of vaccination speed or average daily number of vaccinated people is used; that is, the ratio between the total number of those vaccinated and the number of days that have elapsed since the start of vaccination, choosing when this ratio is at its maximum for each country. By making this choice, we minimize any supply restriction, although it does raise the problem that we fail to consider the same date for all countries.

Method II) involves estimating the model month by month and checking how the significance of the coefficients of the different variables evolves. In principle, this method allows us to know the effect of altruism with and without supply restrictions as well as the time consistency of the results, although the problem is that this method is influenced by changes in health policy and by the course of the disease and the virus.

### Model specification

The specification to be estimated is given by the following:

1 $${V}_{i}={\beta }_{0}+{\beta }_{1}{T}_{i}+{\beta }_{2}{X}_{i}+{\varepsilon }_{i}$$

where *V*_*i*_ indicates the percentage of the population that is fully vaccinated in country i on the corresponding date, and *T*_*i*_ is the representative variable of health altruism in country i. Since we do not have any type of survey that can give us an indicator of this altruism, we use the number of organ transplants in each country as a proxy, since they are the revealed expression of altruism at the highest level in health. Additionally, the vector *X*_*i*_ includes two demand variables that we introduce as control variables: political polarization due to the relationship between antivaccine movements and political extremism and a dummy that identifies the country's former membership in the Eastern Bloc to capture possible public sector distrust in those countries. As explained above, this specification is estimated using data on the day of maximum vaccination speed for each country in Method I and data on vaccination in each month in Method II.

Alternatively, as a robustness test, we estimate the same specification using both methods and substituting the variable transplants (Ti) by the variable organ donations (Di), in order to check that there are no problems due to possible cross-border transfers of organs that may distort the validity of the proxy variable.

### Data sources

The variables employed in the regression analysis to determine how altruism affects vaccination rates and their data sources are described below.

As the dependent variable, we use vaccination rates by country and day (*Vaccination rate*), expressed as the percentage of the population that are fully vaccinated, which is obtained from the Our World in Data database [73].

For those countries that, during the period studied, published vaccination data less frequently than on a daily basis or did not publish data on weekends, as well as for the days on which a country published no data, we linearly interpolate between data from the previous day and the following day to obtain daily frequency time series for each country without any missing values. This solution is impossible to apply to Bulgaria, since during a large part of the study period, Bulgaria reported its data with a very low frequency, reaching periods of over three weeks without reporting new data. Given the impossibility of linear interpolation, we chose to exclude Bulgaria from this study. Finally, for Portugal and Cyprus, no data were given for April, such that the level of vaccination in March is considered.

**Fig. 1:**
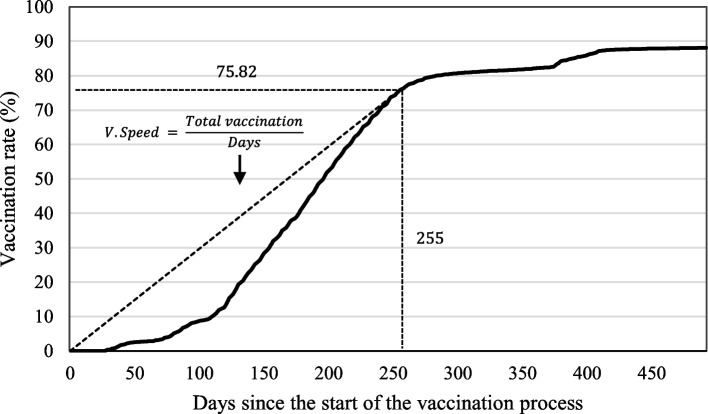
Graphical example of determining the maximum vaccination speed, using the vaccination rate against COVID-19 in Spain. Source: authors’ own compilation based on data from Mathieu, E., Ritchie, H., Ortiz-Ospina, E. et al. A global database of COVID-19 vaccinations. Nat Hum Behav (2021)

In the case of Method I, prior to selecting the values for the dependent variable, the variable we termed vaccination speed must be defined (*Vaccination Speed*). We define vaccination speed, or the average number of individuals vaccinated per day, as the ratio between the total number of people vaccinated and the number of days that have elapsed since the start of the vaccination process. Graphically, if we represent the time series of the accumulated number of vaccinated individuals over time, vaccination speed is represented by the slope of the radius vector that connects the origin (0,0) with any point of the time series representing the number of individuals vaccinated. From this series, we select the data point where the vaccination speed reaches its maximum (see Fig. 1), since at this point, the health system reaches its maximum vaccination capacity, and we can therefore consider that there is no longer any supply restriction affecting the number of people vaccinated. In other words, by choosing the number of people vaccinated on the date when the maximum vaccination speed is reached, we are using the value of the number of vaccinated individuals in the absence of supply restrictions and, therefore, minimizing the incidence of singular logistical factors in each country.

The date we use as a starting point is 8 December 2020, the day on which vaccination began in the EU, specifically in Denmark. We use the same day for the other countries because vaccines were available simultaneously for all of them, since purchasing and distribution of vaccines was centralized. Therefore, any difference in the start of the vaccination process is not related to the provision of vaccines; in other words, it is not caused by supply factors. In the case of Method II, we use data on vaccination rates corresponding to Day 7 of each month starting from the following month; in other words, one month after the start of vaccination.

The main explanatory variable whose impact we wish to test is altruism. We use organ transplants (organ donations as a robustness test) in 2020—the last year available—expressed in terms of the total number of transplants (or donations) of any type as a percentage of the total population as a proxy variable for altruism. Data are extracted from the Global Observatory on Donation and Transplantation (GODT) database (http://www.transplant-observatory.org/). This is the most comprehensive and reliable source regarding donations and transplants. The figures are confirmed by official institutions in each country, and the observatory is coordinated by the WHO and the Spanish Transplant Organization (ONT).

As control variables, taking into account the small number of observations, we include the following:

-An index of political polarization (*Polarization Index*) calculated by aggregating four polarization indices: ideological and affective polarization of the masses and ideological and affective polarization of the elite. This index takes value 10 for the maximum level of political polarization and 0 for the minimum level of political polarization in a given country. The method for calculating the indices is described in the supplementary documentation [74]. The database is available online at https://drive.google.com/drive/folders/1sErZ3Ib-Z3eEr_zXqJ68PWnI-ksVxVxU, and the data used are from 2020, the latest available [74].

- A dummy variable (*Former Eastern Bloc*), which takes the value of one for European Union countries that belonged to the Eastern Bloc (including countries that once belonged to the former Yugoslavia) and zero for the rest.

All the data used in this research are available in the Research Depository of the University of Seville (https://idus.us.es/handle/11441/134624).

## Results

Table 1 shows the main descriptive statistics of the variables used in the estimates, and Table 2 shows the correlation matrix of the explanatory variables. Given the low correlation among variables, we can discard problems related to multicollinearity.

**Table 1:** Descriptive statistics of the variables used to estimate the effect of altruism on vaccination rates against COVID-19 in European Union countries

Variables	(1)	(2)	(3)	(4)	(5)
	**N**	**Mean**	**sd**	**min**	**Max**
Maximum vaccination speed (%)	26	56.09	14.55	21.50	84.68
Vaccination rate 7jan21 (%)	26	0.40	0.40	0.00	1.60
Vaccination rate 7feb21 (%)	26	2.97	0.83	0.98	5.69
Vaccination rate 7mar21 (%)	26	7.21	1.79	3.51	12.59
Vaccination rate 7apr21 (%)	26	15.22	4.79	7.68	31.87
Vaccination rate 7may21 (%)	26	28.15	6.81	17.18	49.53
Vaccination rate 7jun21 (%)	26	41.70	7.75	23.26	63.75
Vaccination rate 7jul21 (%)	26	52.13	10.62	25.02	71.13
Vaccination rate 7ago21 (%)	26	59.15	12.58	26.48	78.36
Vaccination rate 7sep21 (%)	26	63.27	13.80	27.63	86.56
Vaccination rate 7oct21 (%)	26	65.37	13.47	29.24	88.29
Vaccination rate 7nov21 (%)	26	67.47	12.85	31.23	88.90
Vaccination rate 7dec21 (%)	26	69.70	12.17	33.29	90.27
Vaccination rate 7jan22 (%)	26	71.42	12.33	35.55	92.78
Vaccination rate 7feb22 (%)	26	72.95	12.59	37.98	94.78
Vaccination rate 7mar22 (%)	26	73.32	12.45	40.31	95.04
Vaccination rate 7apr22 (%)	26	73.55	12.26	42.69	95.04
Polarization Index	26	4.64	0.49	3.54	5.65
Organ donations (%)	26	1.67e-05	8.72e-06	3.42e-06	3.75e-05
Transplants (%)	26	1.58e-05	7.96e-06	3.42e-06	3.34e-05
Former Eastern Bloc	26	0.38	0.50	0.00	1.00

Source: authors’ own compilation

**Table 2:** Estimation of the maximum speed of vaccination against COVID-19 in the countries of the European Union

Country	Day when maximum vaccination speed is reached (1)	Number of days since 12/08/2020 until reaching maximum vaccination speed (2) = (1) –12/8/20	Total number of those vaccinated until the day of maximum speed (number of vaccinated) (3)	Vaccination level on the day of maximum speed (% people over total) (4)	Maximum vaccination speed (number of vaccinated / number of days) (5) = (3)/(2)	Total population (millions) (6)	Maximum vaccination rate (% of the total population / number of days) (7) = (3) / [(5)·(2)]
Austria	02/07/2021	207	4,971,228	54.97	24,015.59	9,043,072	0.2656
Belgium	03/07/2021	208	7,470,771	64.22	35,917.17	11,632,334	0.3088
Check Republic	25/06/2021	200	4,986,583	46.50	24,932.92	10,724,553	0.2325
Croatia	11/06/2021	186	1,401,647	34.34	7,535.74	4,081,657	0.1846
Cyprus	17/06/2021	192	443,121	49.46	2,307.92	896,005	0.2576
Denmark	29/07/2021	234	4,149,738	71.38	17,733.92	5,813,302	0.3051
Estonia	18/06/2021	193	540,086	40.76	2,798.37	1,325,188	0.2111
Finland	04/07/2021	209	3,380,819	60.93	16,176.17	5,548,361	0.2915
France	13/08/2021	249	46,673,270	69.23	187,442.85	67,422,000	0.2780
Germany	09/07/2021	214	49,023,479	58.43	229,081.68	83,900,471	0.2730
Greece	23/07/2021	228	5,578,596	53.79	24,467.53	10,370,747	0.2359
Hungary	22/05/2021	166	5,005,682	51.96	30,154.71	9,634,162	0.3130
Ireland	04/08/2021	240	3,460,704	69.45	14,419.60	4,982,904	0.2894
Italy	02/07/2021	207	34,862,781	57.75	168,419.23	60,367,471	0.2790
Latvia	17/11/2021	345	1,250,025	66.96	3,623.26	1,866,934	0.1941
Lithuania	20/08/2021	256	1,607,299	59.75	6,278.51	2,689,862	0.2334
Luxemburg	17/07/2021	222	384,752	60.61	1,733.12	516,100	0.2730
Malta	25/05/2021	169	310,403	60.14	1,836.70	634,814	0.3559
Netherlands	10/07/2021	215	11,691,199	68.08	54,377.67	17,173,094	0.3166
Poland	18/06/2021	193	16,045,779	42.45	83,138.75	37,797,000	0.2200
Portugal	29/08/2021	265	8,610,175	84.68	32,491.23	10,167,923	0.3195
Romania	22/05/2021	166	4,104,686	21.46	24,727.02	19,127,772	0.1293
Slovakia	31/05/2021	175	1,811,015	33.16	10,348.66	5,460,726	0.1895
Slovenia	18/06/2021	193	814,831	39.20	4,221.92	2,078,723	0.2031
Spain	19/08/2021	255	35,442,173	75.82	138,988.91	46,745,211	0.2973
Sweden	25/07/2021	230	6,253,678	61.55	27,189.90	1,016,0159	0.2676

Source: authors’ own elaboration based on data from Mathieu, (2021) [75]

In addition, after analysing the correlation matrix of the independent variables used in the estimates, whose results are available in the supplementary material, we verify that the models do not present multicollinearity problems, since the correlation between variables is low.

### Influence of health altruism on vaccination against COVID-19, Method I

Table 2 show the data corresponding to the date of maximum vaccination rate.

Finally, Table 3 collects the estimated specification.

**Table 3:** Estimated influence of health altruism on the vaccination rate of EU countries using the vaccination rate corresponding to the day of maximum vaccination speed in each EU country as the dependent variable

	**Vaccination rate**
**Demand variables**	**Coefficients**
**Transplants**	637,872**
	(243,583)
**Polarization Index**	-6.765*
	(3.428)
**Former Eastern Bloc**	-19.09***
	(4.574)
**Constant**	84.76***
	(14.00)
**Observations**	26
**R-square**	0.595

Source: original elaboration based on data from Mathieu, (2021) [75]. Note: robust standard errors in parentheses

^***^p < 0.01, **p < 0.05, *p < 0.1

The results show that the representative variable of health altruism positively influences the vaccination rate, thereby confirming our hypothesis. Likewise, the other two demand variables, political polarization and the country’s membership in the Eastern Bloc, work against vaccination and are statistically significant.

The results shown in Table 3 suggest the need to assess the possible relationship between the final vaccination rate and the maximum vaccination speed and the time until maximum speed is reached. Table 4 shows the results of the analysis of this relationship. These results show that the longer the period and the greater the maximum vaccination speed are, the higher the final vaccination rate. Both factors explain almost 90% of the final vaccination rate value.

**Table 4:** Relationship between maximum vaccination speed and days since the start of vaccination in the European Union until the day the maximum vaccination speed is reached with the final level of vaccination by country. The dependent variable is the final vaccination rate

	**Vaccination rate**
Variable	**Coefficients**
Constant	-2.1046 (4.4011)
Days to maximum speed	0.1163*** (0.0267)
Maximum vaccination speed	195.4141*** (17.2801)
Observations	26
R-square	0.8845

Source: own elaboration. Notes: OLS regression. Robust standard errors in parentheses

^***^p < 0.01, **p < 0.05, *p < 0.1

### Influence of health altruism on vaccination against COVID-19, method II

Table 5 shows the results obtained. During the first phase of vaccination, when supply constraints are in effect, demand variables have no effect. However, when supply restrictions disappear and individuals who want to be vaccinated begin to weigh more, these variables become relevant.

**Table 5:** Estimation of the impact of health altruism on the vaccination rate of EU countries corresponding to each month since the start of vaccination on 8 December 2020

	Vaccination rate							
Date	(1)	(2)	(3)	(4)	(5)	(6)	(7)	(8)
	7/1/21	7/2/21	7/3/21	7/4/21	7/5/21	7/6/21	7/7/21	7/8/21
Transplants	13,808	-19,574	-13,254	-50,554	-48,055	12,386	243,583	440,820**
	(9,009)	(17,617)	(39,004)	(117,786)	(175,916)	(201,968)	(195,321)	(189,993)
Polarization Index	-0.160	-0.396	-1.073	-2.339	-2.573	-2.331	-4.206	-5.857*
	(0.197)	(0.424)	(0.944)	(2.840)	(3.811)	(3.998)	(3.267)	(3.195)
Former Eastern Bloc	0.122	-0.392	-0.651	-0.832	-4.146	-7.637**	-15.91***	-19.86***
	(0.152)	(0.328)	(0.696)	(1.896)	(2.627)	(2.765)	(2.796)	(2.796)
Constant	0.879	5.263**	12.65**	27.19*	42.45**	55.25***	73.93***	87.01***
	(0.869)	(2.265)	(4.905)	(14.25)	(18.52)	(19.15)	(14.39)	(14.14)
Observations	26	26	26	26	26	26	26	26
R-square	0.091	0.181	0.141	0.087	0.144	0.274	0.641	0.756

	Vaccination rate							
Date	(9)	(10)	(11)	(12)	(13)	(14)	(15)	(16)
	7/9/21	7/10/21	7/11/21	7/12/21	7/1/22	7/2/22	7/3/22	7/4/22
Transplants	599,948***	600,481***	569,776**	545,537**	521,730**	468,392**	436,847**	414,240**
	(201,950)	(203,854)	(207,967)	(209,018)	(206,180)	(203,250)	(190,999)	(177,106)
Polarization Index	-6.154*	-5.878	-6.368*	-6.114*	-6.164*	-5.768*	-5.596*	-5.434*
	(3.423)	(3.454)	(3.400)	(3.197)	(3.161)	(3.252)	(3.207)	(3.121)
Former Eastern Bloc	-21.20***	-20.55***	-18.94***	-17.87***	-18.31***	-19.29***	-19.30***	-19.20***
	(3.044)	(2.996)	(3.127)	(2.986)	(3.006)	(2.998)	(2.921)	(2.836)
Constant	90.50***	91.08***	95.31***	96.33***	98.83***	99.74***	99.81***	99.61***
	(15.04)	(15.14)	(14.74)	(13.57)	(13.49)	(14.27)	(14.39)	(14.30)
Observations	26	26	26	26	26	26	26	26
R-square	0.755	0.751	0.720	0.719	0.720	0.726	0.730	0.737

Source: original elaboration based on data from Mathieu, (2021) [75]. Note: robust standard errors in parentheses

*** *p* < 0.01, ** *p* < 0.05, * *p* < 0.1

Estimates by month throughout the period in which vaccination took place show a positive relationship between health altruism and vaccination rate, starting from the period in which restrictions on the supply of vaccines began to disappear and the vaccine began to be fully accessible (eighth month of vaccination). In the same period, the estimates capture the influence of political polarization and being a former Soviet Bloc country. In the latter case, the negative relationship appeared a couple of months earlier. As seen in the table, the results are robust throughout the period, both in the value of the estimated coefficients and in their statistical significance.

We check the robustness of our results by estimating both methods using organ donations instead of transplants. The results, included in the Supplementary Information, are similar to the baseline and allow us to verify there are no problems related to cross-border transfers of organs that may distort the validity of the proxy variable.

## Discussion

The results obtained show that individuals’ altruism, measured through a proxy variable such as transplants per capita, favours vaccination against COVID-19. The results are robust and are supported by the two methodologies used.

### The role of health altruism in vaccination

Countries that traditionally show a more committed attitude towards the health of others, that is, countries that value the external effects of their behaviour and demonstrate this through a greater number of transplants, have achieved higher rates of vaccination against COVID-19. When we take the vaccination rate at the time of the highest vaccination speed, the value of the coefficient is greater than 600, and when we perform monthly estimates, the significance of the coefficient is maintained over time from the eighth month of vaccination, although the value of its coefficient begins to decrease from the eleventh month. This evolution of the estimated coefficient could be related to a) the loss of altruism weight as vaccination progresses and people are already vaccinated; b) the course of the pandemic, since the appearance of the Omicron variant caused the vaccination processes to accelerate again due to nonvaccinated people’s fear of becoming infected; and c) because European governments began to require vaccination passports for work and leisure [49]. In this sense, further research is needed.

### The impact of other demand and supply factors

On the opposite side of altruism, there are the other two demand factors included in the specification that work against vaccination. On the one hand, the dummy variable representing a country’s membership in the Eastern Bloc shows that these countries have vaccination rates that are approximately 19% lower, which confirms previous results [76] that already anticipated the negative effect of this condition as a consequence of elderly people’s distrust of public policies.

On the other hand, political polarization, which is also related to political mistrust, eventually manifests itself in antivaccine positions and lower vaccination rates, which is also captured by our estimation using the two methods [77]. The relationship between electoral support for populist political parties and rejection of vaccination was already evident before the COVID-19 pandemic and shows how political populism and vaccine rejection are driven by similar dynamics: deep mistrust towards elites and experts. Later, during the pandemic, political extremism reduced the population’s willingness to comply with social distancing measures [59] and caused the rejection of vaccination [15], a result that our work also confirms.

Regarding supply factors, as expected, with Method I), by assessing vaccination at the time of maximum speed or average daily vaccination, we were able to isolate the effects of these supply factors, as was our intention. However, as we observed with Method II), supply factors would have had an impact until the sixth month of vaccination, and the estimate does not stabilize until the eighth month of vaccination. We must understand that this is because, in that period, the EU still lacked sufficient vaccines and that it was therefore the supply shortage that affected vaccination.

### Limitations

This work has limitations that must be taken into account. First, we must consider the restricted geographical area. We only have 26 observations in each estimate, which limited the possibility of expanding the number of variables. The negative effect of this reduced number of observations was offset by removing the effect of supply factors, yet any replication of the work for larger geographical areas will require these variables to be introduced to control for economic and logistical constraints in countries with lower incomes per capita.

In addition, this work does not control for the impact of the course of the disease itself (new variants of SARS-CoV-2, waves of contagion, etc.) or the measures adopted to control the spread of the disease. Both impact how vaccination progressed, and it is therefore reasonable to assume that they would alter the relevance of altruism in vaccination. In fact, the variation in the coefficients estimated from month 12 for the proxy variable is very likely related to this issue.

### Implications

Important implications emerged from the results. First, we observed that during the period of supply shortage, the fundamental objective was to eliminate this shortage, since the other factors were not relevant for vaccination if production was not capable of meeting demand. Once supply constraints began to ease, demand factors came into play, and altruism favoured vaccination. For this reason, health authorities should promote vaccination, for example, through advertising campaigns to raise public awareness that influence health altruism and disseminate the benefits of vaccination for the health of people around us.

In addition, given that countries where health altruism is more developed have an initial advantage in terms of vaccination processes, health authorities must invest proactively and systematically to promote such health altruism. In particular, governments should value fostering health altruism in education since this can yield high social returns in the long term when we are faced with new pandemics.

Another implication of the results is the need to prevent rejection of vaccination from radical political positions. Governments must be aware that political radicalism operates against vaccination, and they must work preventively by drawing up contingency plans to reduce antivaccine reactions in radicalized countries. In this sense, it may be important to promote agreements between parties vis-à-vis leaving vaccines out of the political dispute and establishing regulations that make vaccination mandatory or that sanction nonvaccination (prohibiting access to workplaces and the hospitality/catering sector for unvaccinated people or mandatory vaccination passports). These actions must be planned in advance and must take effect at a time when there is no shortage of vaccines and when the speed of vaccination continues to increase.

This last recommendation—that governments should focus their efforts on the first phase of vaccination, that is, during the period from the start of the vaccination process until the point when the maximum vaccination speed is reached—is deduced from the relationship of the final vaccination rate with the maximum vaccination speed and the time elapsed until it is reached. As seen in the estimate shown in Table 4, both variables are positively correlated with the final level of vaccination, so we can conclude that this period is of utmost importance for the final situation of the country in terms of the vaccination rate.

Finally, it is necessary to reflect on Eastern European countries and their particularity in terms of vaccination. The effects of low vaccination rates against COVID-19 manifest themselves in an excess mortality of close to 30% [78], coinciding with vaccination rates that, as we have seen, were approximately 19% lower. This situation highlights the need to focus on studying these countries and to adopt measures to reverse this mistrust that proves detrimental to public health.

In summary, bearing in mind that pandemics such as COVID-19 might happen again, the study concludes that governments must plan actions to favour vaccination by systematically promoting health altruism in the long term. These actions guarantee a good starting point for possible future pandemic contexts. In addition, it is also necessary to plan positive and negative incentive processes for vaccination in a pandemic –focusing on the first phase of vaccination– so as to reduce the negative impact of the factors that act against vaccination.

## Conclusions

This article tests the hypothesis that health altruism has a positive impact on population vaccination against Covid-19. We represent altruism in health through a proxy variable –organ transplants– to reflect the greater acceptance of vaccination when individuals assume the positive external effects for their direct relatives and for society in general, by helping to achieve herd immunity.

The results obtained confirm the following hypothesis:

- First, that health altruism is an explanatory factor of demand, and positively influences countries’ vaccination rates.
- Second, there are other demand factors that work against vaccination, such as the country’s political polarization or being a former Eastern Bloc country.

Both conclusions point to the need to continue researching the incidence of demand factors in vaccination and, especially, the usefulness of promoting healthcare altruism in the population in the face of future pandemic.

## Supplementary Information

**Additional file 1: Appendix A.** Evolution of vaccination across European Union countries. **Appendix B.** Results using organ donations as proxy for altruism. **Appendix C.** Description of the Polarization Index. **Appendix D.** Results of panel data model. **Appendix E.** Correlation matrix of independent variables.

**Additional file 2. **Data.

## Data Availability

The datasets are available as Supplementary Information of this article and can be found at the University of Seville’s data repository. The URL for the dataset is: https://hdl.handle.net/11441/134624.

## Abbreviation

EU: European Union
