# Supplementary material for: The effect of altruism on COVID-19 vaccination rates
Source: Health Econ Rev. 2023 Jan 3;13:2. doi: 10.1186/s13561-022-00415-6 (PMC9807973; doi:10.1186/s13561-022-00415-6)
Supplement: Supplementary file 1 — Additional file 1: Appendix A. Evolution of vaccination across European Union countries. Appendix B. Results using organ donations as proxy for altruism. Appendix C. Description of the Polarization Index. Appendix D. Results of panel data model. Appendix E. Correlation matrix of independent variables. [file 13561_2022_415_MOESM1_ESM.docx]

# The effect of altruism on COVID-19 vaccination rates^[[1]](#footnote-1)^

Luis Ángel Hierro^1^

David Patiño^1^

Pedro Atienza^1^

Antonio José Garzón^1^

David Cantarero^2^

^1^*University of Seville*

^2^*University of Cantabria*

**Supplementary Information**

**Additional file 1**

**Appendix A. Evolution of vaccination across European Union countries**

**Figure A1.** Evolution of the vaccination rate in the European Union’s countries.

*Note:* The black line denotes the country’s vaccination rate and the red dashed line represents the EU’s weighted average vaccination rate.

**Figure A1** (continued). Evolution of the vaccination rate in the European Union’s countries.

*Note:* The black line denotes the country’s vaccination rate and the red dashed line represents the EU’s weighted average vaccination rate.

**Figure A1** (continued). Evolution of the vaccination rate in the European Union’s countries.

*Note:* The black line denotes the country’s vaccination rate and the red dashed line represents the EU’s weighted average vaccination rate.

**Appendix B. Results using organ donations as proxy for altruism.**

**Table B1**. Estimated influence of health altruism on the vaccination rate of European Union countries corresponding to the day of maximum vaccination rate in each country.

| **Demand variables** | **Vaccination rate**  **Coefficients** |
| --- | --- |
| Donations | 564,745** |
|  | (221,907) |
| Polarization Index | -6.700* |
|  | (3.545) |
| Former Eastern Bloc | -19.19*** |
|  | (4.611) |
| Constant | 85.15*** |
|  | (14.57) |
|  |  |
| Observations | 26 |
| R-square | 0.588 |

Source: original elaboration based on data from Mathieu, (2021) [70]. Note: robust standard errors in parentheses. *** p<0.01, ** p<0.05, * p<0.1

**Table B2.** Estimation of the incidence of health altruism in the vaccination rate of the countries of the European Union corresponding to each month since the start on December 8, 2020.

|  | **Vaccination rate** | | | | | | | |
| --- | --- | --- | --- | --- | --- | --- | --- | --- |
| **Date** | **(1)**  **7/1/21** | **(2)**  **7/2/21** | **(3)**  **7/3/21** | **(4)**  **7/4/21** | **(5)**  **7/5/21** | **(6)**  **7/6/21** | **(7)**  **7/7/21** | **(8)**  **7/8/21** |
|  |  |  |  |  |  |  |  |  |
| Donations | 13,402 | -18,220 | -12,703 | -50,668 | -52,442 | 3,871 | 205,373 | 381,946** |
|  | (8,345) | (15,754) | (34,770) | (105,774) | (159,546) | (183,466) | (180,988) | (178,021) |
| Polarization Index | -0.166 | -0.393 | -1.069 | -2.308 | -2.517 | -2.286 | -4.118 | -5.761* |
|  | (0.197) | (0.426) | (0.950) | (2.855) | (3.831) | (4.019) | (3.286) | (3.248) |
| Former Eastern Bloc | 0.121 | -0.390 | -0.650 | -0.832 | -4.151 | -7.648** | -15.96*** | -19.94*** |
|  | (0.151) | (0.327) | (0.695) | (1.892) | (2.625) | (2.774) | (2.824) | (2.844) |
| Constant | 0.900 | 5.241** | 12.63** | 27.09* | 42.31** | 55.18*** | 73.96*** | 87.19*** |
|  | (0.872) | (2.260) | (4.901) | (14.24) | (18.53) | (19.16) | (14.37) | (14.25) |
|  |  |  |  |  |  |  |  |  |
| Observations | 26 | 26 | 26 | 26 | 26 | 26 | 26 | 26 |
| R-square | 0.099 | 0.182 | 0.142 | 0.088 | 0.146 | 0.274 | 0.637 | 0.749 |

|  | **Vaccination rate** | | | | | | | |
| --- | --- | --- | --- | --- | --- | --- | --- | --- |
| **Date** | **(9)**  **7/9/21** | **(10)**  **7/10/21** | **(11)**  **7/11/21** | **(12)**  **7/12/21** | **(13)**  **7/1/22** | **(14)**  **7/2/22** | **(15)**  **7/3/22** | **(16)**  **7/4/21** |
|  |  |  |  |  |  |  |  |  |
| Donations | 526,802** | 534,366*** | 505,526** | 487,705** | 469,353** | 421,518** | 393,017** | 372,462** |
|  | (189,287) | (188,959) | (192,131) | (191,695) | (187,386) | (184,499) | (173,367) | (160,792) |
| Polarization Index | -6.066* | -5.834 | -6.316* | -6.087* | -6.156* | -5.762* | -5.590* | -5.427* |
|  | (3.493) | (3.515) | (3.462) | (3.251) | (3.211) | (3.287) | (3.238) | (3.150) |
| Former Eastern Bloc | -21.29*** | -20.64*** | -19.02*** | -17.94*** | -18.38*** | -19.35*** | -19.36*** | -19.25*** |
|  | (3.111) | (3.040) | (3.178) | (3.035) | (3.050) | (3.037) | (2.956) | (2.868) |
| Constant | 90.82*** | 91.47*** | 95.67*** | 96.72*** | 99.23*** | 100.1*** | 100.1*** | 99.93*** |
|  | (15.25) | (15.34) | (14.94) | (13.77) | (13.69) | (14.39) | (14.48) | (14.37) |
|  |  |  |  |  |  |  |  |  |
| Observations | 26 | 26 | 26 | 26 | 26 | 26 | 26 | 26 |
| R-square | 0.746 | 0.745 | 0.714 | 0.714 | 0.717 | 0.723 | 0.728 | 0.735 |

Source: Original elaboration based on data from Mathieu, (2021) [70]. Note: robust standard errors in parentheses. *** p<0.01, ** p<0.05, * p<0.1.

**Appendix C. Description of the Polarization Index**

The Polarization Index used in this work follows the index developed by van der Veen (2021) and is calculated as the average of four types of polarization: ideological polarization of the elites, ideological polarization of the masses, affective polarization of the elites and affective polarization of the masses. Therefore, its formula is as follows:

|  | ${IP}_{s}= \frac{{EI}_{s}+{MI}_{s}+{EA}_{s}+{MA}_{s}}{4}$ | (C1) |
| --- | --- | --- |

Where ${EI}_{s}$ is the index of ideological polarization of the elites, ${MI}_{s}$ is the index of ideological polarization of the masses, ${EA}_{s}$ is the index of affective polarization of the elites and ${MA}_{s}$ is the index of affective polarization of the elites. The suffix *s* refers to each country in the sample. Data employed to calculate these indicators are obtained from different surveys: CSES, Latinobarómetro, WVS, Afrobarometer, CHES MARPOR and VDem.

To calculate the ideological polarization of the elites, we use the ideological distance between the parties from the country, weighted by their votes. This measure is normalized so that it takes values between 0 and 10, with 0 being equal ideologies between the parties and 10 being the maximum polarization. Its formula is as follows:

|  | ${EI}_{s}= \sqrt{\sum_{i=1}^{n} p_{i} x \left( \frac{I_{i}-\bar{I}_{i}}{5} \right)^{2}}$ | (C2) |
| --- | --- | --- |

Where $p_{i}$ is the proportion of votes obtained by party i, $I_{i}$ is the ideological position of the party and $\bar{I}_{i}$ is the average ideological position in the country.

Regarding the ideological polarization of the masses, we employ an approach similar to the previous index, although, in this case, at the level of individuals instead of political parties. Finally, van der Veen (2021) makes a transformation so that the indicator has a range from 0 to 10 and is comparable with the rest of indicators.

|  | ${MI}_{s}= \sqrt{\sum_{i=1}^{n} p_{i} \frac{\left( \frac{I_{i}-\bar{I}_{i}}{5} \right)^{2}}{n}}$ | (C3) |
| --- | --- | --- |

In this case, $p_{i}$ represents the proportion of individuals who share certain ideological position $I_{i}$.

For the affective polarization of the elites, the data is obtained directly from the VDem survey in which experts are consulted about this concept for each country and year. However, the values have a range from -3.5 to 3.5, so they are normalized to a range of 0 to 10 to be comparable and to be aggregated.

Finally, to calculate the affective polarization of the masses, we use the results per individual obtained from the CSES survey and added at the country level following the methodology of Lauka, McCoy and Firat (2018). The formula is the following:

|  | ${MA}_{s}=80 x \left( \frac{\sum_{i=1}^{np} Lp_{i} x {Dp}_{i}}{n} \right)$ | (C4) |
| --- | --- | --- |

Where $Lp_{i}$ is the proportion of parties to which individuals show a positive feeling and $Dp_{i}$ the proportion of parties to which individuals show a negative considerations.

**Appendix D. Results of panel data model**

In our baseline specification, we estimate the relationship between the vaccination rate and its explanatory variables through a sequence of cross-section regressions for each month since the start of the vaccination process. In this section, we do the estimates using panel data regressions, that is, we include the temporal dimension to the previously analysed spatial dimension. However, it is necessary to take into account that the explanatory variables, both altruism and the control variables, remain invariant throughout the sample, so we are fored to use the random effects method to avoid multicolinearity issues. The specification that we estimate is the following:

|  | $V_{i,t}=\beta_{0}+\beta_{1}T_{i,t}+\beta_{2}X_{i,t}+\varepsilon_{i,t}$ | (D1) |
| --- | --- | --- |

Where $V_{i,t}$ indicates the percentage of the population fully vaccinated in country i in month t and $T_{i,t}$ is the proxy variable of healthcare altruism in country i and period t. For its part, the vector $X_{i,t}$ is set of demand variables that we introduce as control variables: political polarization and a dummy that identifies the country's former membership of the Eastern bloc, to capture possible distrust of the public sector in those countries. The results are shown in table D1.

**Table D1**. Estimation of the impact of health altruism (Transplants and Organ donations) on the vaccination rate of EU countries using panel data.

|  | Vaccination rate | | | | |
| --- | --- | --- | --- | --- | --- |
|  | **(1)** | **(2)** |  | **(3)** | **(4)** |
| Variables | **OLS** | **Random Effects** | **Variables** | **OLS** | **Random Effects** |
|  |  |  |  |  |  |
| Transplants | 296,007 | 296,007** | **Donations** | 261,332 | 261,332** |
|  | (184,400) | (134,982) |  | (169,604) | (124,068) |
| Polarization Index | -4.151 | -4.151* | **Polarization Index** | -4.116 | -4.116* |
|  | (3.238) | (2.445) |  | (3.245) | (2.470) |
| Former Eastern Bloc | -12.75*** | -12.75*** | **Former Eastern Bloc** | -12.79*** | -12.79*** |
|  | (2.657) | (1.880) |  | (2.656) | (1.908) |
| Constant | 67.24*** | 67.24*** | **Constant** | 67.41*** | 67.41*** |
|  | (14.63) | (11.20) |  | (14.65) | (11.24) |
|  |  |  |  |  |  |
| Observations | 416 | 416 | **Observations** | 416 | 416 |
| R-squared | 0.059 |  | **R-squared** | 0.059 |  |
| Number of country |  | 26 | **Number of country** |  | 26 |

Source: authors’ own compilation. Robust standard errors in parentheses. *** p<0.01, ** p<0.05, * p<0.1.

These results confirm those obtained in our base specification. That is, using both Donations and Transplants as a proxy for altruism, we find that they have a positive impact on the country's vaccination rate, while both polarization and membership of the former Eastern bloc maintain a negative relation with the country’s vaccination rate.

**Appendix E. Correlation matrix of independent variables**

**Table E1**. Correlation matrix of the explanatory variables used to estimate the effect of altruism in the vaccination rate against COVID-19 in European Union countries.

|  | **Transplants** | **Polarization Index** | **Former Eastern Bloc** |
| --- | --- | --- | --- |
| **Transplants** | 1.000 |  |  |
| **Polarization Index** | 0.329 | 1.000 |  |
| **Former Eastern Bloc** | -0.045 | 0.100 | 1.000 |

Source: authors’ own compilation.

1. [↑](#footnote-ref-1)
